# Scaling Access to Contraception for Youth in Urban Slums: The Challenge Initiative's Systems-Based Multi-Pronged Strategy for Youth-Friendly Cities

**DOI:** 10.3389/fgwh.2021.673168

**Published:** 2021-10-01

**Authors:** Krishna Bose, Kim Martin, Kathryn Walsh, Maheen Malik, Paul Nyachae, Morine Lucy Sierra, Albert Bwire, Denis Sama, Tumaini Kiyola, Vanessa Mitchell, Hawa Talla, Josephat Avoce, Kate Graham, Mukesh Sharma, Devika Varghese, Andrea Ferrand, Victor Imuwahen Igharo, Dorcas Nelson Akila

**Affiliations:** ^1^Bill and Melinda Gates Institute for Population and Reproductive Health, Bloomberg School of Public Health, Johns Hopkins University, Baltimore, MD, United States; ^2^Deloitte, New York, NY, United States; ^3^Jhpiego, Nairobi, Kenya; ^4^IntraHealth International, Chapel Hill, NC, United States; ^5^Population Services International, Washington, DC, United States; ^6^Johns Hopkins Center for Communication Programs, Baltimore, MD, United States

**Keywords:** adolescents, sexual and reproductive health, urban slums, contraception, scale, family planning, youth

## Abstract

**Introduction:**

More than half of all adolescents globally live in Asia, with India having the largest adolescent population in the world at 253 million. In sub-Saharan Africa, adolescents make up the greatest proportion of the population, with 23% of the population aged 10–19. And these numbers are predicted to grow rapidly—particularly in urban areas as rural youth migrate to cities for economic opportunities. While adolescents and youth are subject to high sexual and reproductive health risks, few efforts have been documented for addressing these in urban settings, especially in poor settlements.

**Methods:**

The Challenge Initiative (TCI) is a demand-driven, family planning platform for sustainable scale and impact that lets city governments—in particular urban slums—lead implementation. It is currently active in 11 countries in Africa and Asia. In June 2018, TCI heightened its focus on adolescent and youth sexual and reproductive health (AYSRH) for youth living in urban slums. It now supports 39 city governments. TCI dedicates technical and program support to married (including first-time parents) and unmarried youth ages 15–24 years. Using an innovative coaching model and an online learning platform (TCI University), TCI supports city governments as they implement AYSRH interventions to accelerate the impact of TCI's model for rapid scale.

**Results:**

TCI has been assessing the performance of cities implementing its AYSRH approaches using its RAISE tool and has found considerable improvement over two rounds of assessments through TCI coaching and support for adaptation of its high-impact interventions between the first and second round.

**Conclusions:**

TCI's AYSRH approach scaled rapidly to 39 cities and multiple urban slums since 2018, using its evidence-based interventions and coaching model. In the context of universal health coverage, TCI has supported segmented demand generation and improved access to quality and affordable contraceptive as well as youth-friendly health services. It provides a menu of interventions for cities to implement for youth—including such approaches as public-private partnerships with pharmacies and quality assurance using quick checklists—along with an innovative coaching model. This approach has facilitated greater access to contraceptive methods of choice for youth.

## Introduction

Across the world, a disproportionate number of youth are migrating to urban centers to seek better economic opportunities than found in the agricultural sector in rural areas (1, 2). While 56% of the world's population lived in urban areas in 2019 (3), it is estimated that 60% of urban populations will be under the age of 18 by 2030 (4). Currently, more than half of all adolescents live in Asia—with India having the largest adolescent population in the world at 253 million (5). In sub-Saharan Africa, adolescents make up the greatest proportion of the population, with 23% of the population aged 10–19 (6).

While it is recognized that this age group faces high sexual and reproductive health risks (7), few efforts have been documented for addressing these in urban settings, where the situation is further complicated by poverty, population density, and a lack of access to quality services, including reproductive health services (8). Most developing country governments have focused on improving services in rural areas, not taking into consideration the rapid urbanization taking place and burgeoning urban slums with inadequate or non-existent public health services.

Adolescents and youth in urban slums are vulnerable to early unintended pregnancy that result in poor social, health, and economic outcomes not only for young mothers but young fathers too (9). These young mothers and their children are also at higher risk for adverse birth outcomes (10, 11) when compared with older mothers. There are also intergenerational effects for children of young mothers, who tend to have lower educational achievement scores and poorer socioemotional outcomes (12, 13).

Depending on their country, city, socioeconomic status, or gender, adolescents have insufficient, inaccurate, incorrect, or poor knowledge and information about safe sex and contraceptive use. This situation is worsened by the lack of availability of, and access to, adolescent and youth-friendly health services (AYFHS) and health products for adolescents. Thus, many adolescents and youth do not have the means to protect themselves with condoms or other contraceptives or to use health services for prevention, promotion, and maintenance of their good health should they contract a disease (e.g., a sexually-transmitted infection) (14). So while many young women in sub-Saharan Africa are sexually active and would like to avoid pregnancy, contraceptive use remains low with national DHS prevalence data (2010–2014) showing Benin at 10.5%, Kenya 37.7%, Nigeria 12.6%, Senegal 8.1%, Tanzania 23.7%, and Uganda 18.1% (15).

Globally, unmet need for contraception remains relatively constant at 23%, reflecting rising demand. In the developing world, an estimated 38 million girls were sexually active (had intercourse in the past 3 months) and wanted to avoid pregnancy in 2016, but only 15 million reported use of a modern contraceptive, leaving 23 million—or 60%—at risk of unintended pregnancy (16).

Recognizing that adolescent birth rates in sub-Saharan Africa are the highest in the world at 104 births per 1,000 adolescent girls, The Challenge Initiative (TCI) focuses its efforts there on preventing teen and repeat pregnancies (17–19). In India, most young women experience their sexual debut within marriage and are often pressured to bear children. For them, TCI supports interventions after the first child as the most effective strategy. In summary, while sexual activity and unmet need for contraception are common among adolescents, clear differences exist in age, sex, rural/urban region, country, and marital status. Therefore, evidence-based intervention strategies are based on common principles (20), but adapted by each city for their local context.

These emerging evidence-based interventions (21, 22) include addressing provider bias and improving the quality of services to make them youth friendly, implementing strategic behavior change communication campaigns segmented for youth age-cohorts, and involving youth in designing, advocating for and supporting the implementation and monitoring of programs. However, these interventions are not yet widely adopted, suffer from limited funding, and are difficult to scale up.

## Materials and Methods

### Study Setting

**Figure 1:**
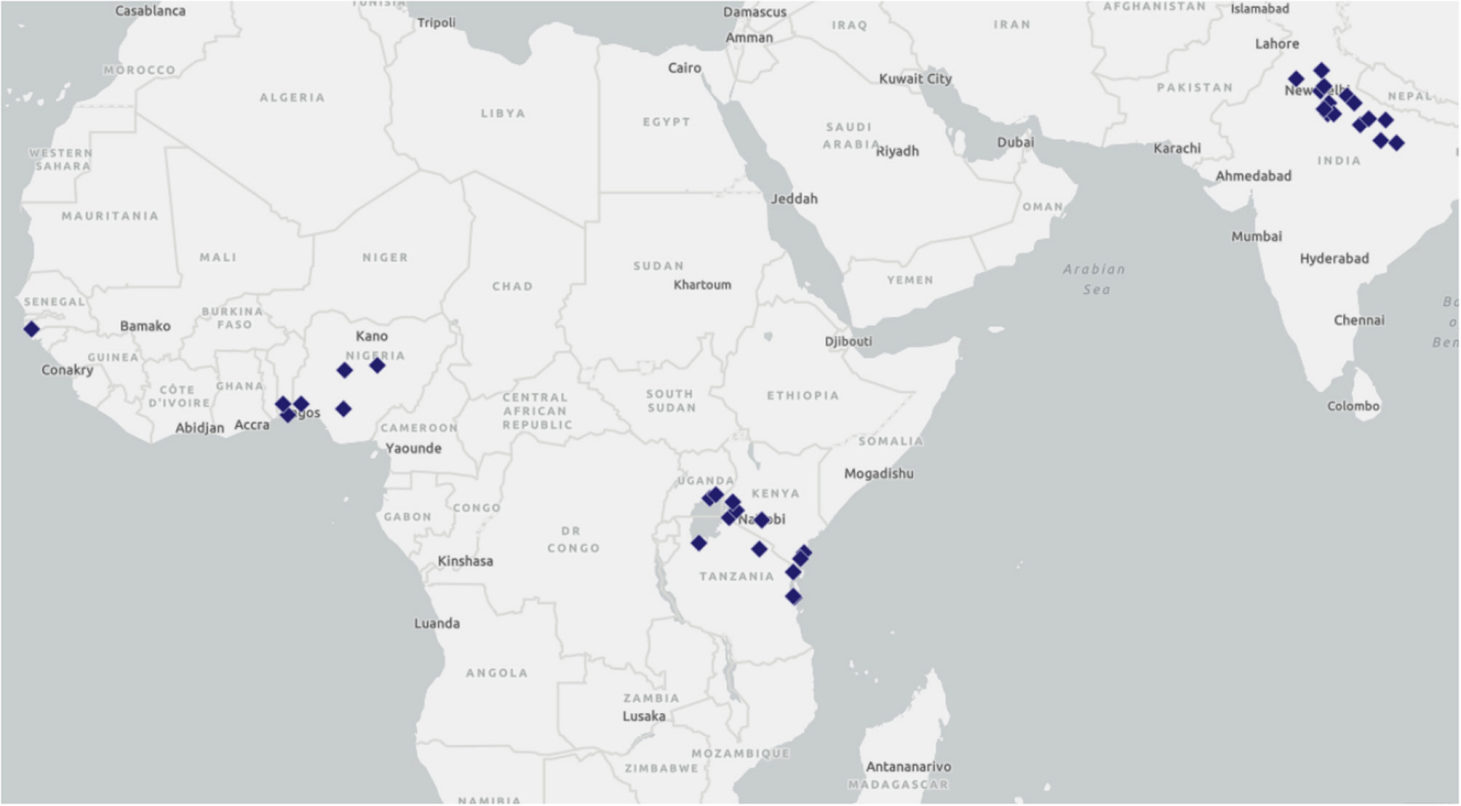
TCI is working with local governments in 39 cities/states across seven countries to implement AYSRH programming.

TCI is a Bill & Melinda Gates Foundation-funded family planning platform currently being implemented by city governments in 11 countries in Africa and Asia (India, Senegal, Benin, Burkina Faso, Cote d'Ivoire, Niger, Nigeria, Kenya, Uganda, Tanzania, and Philippines). The Gates Institute for Population and Reproductive Health (GI), housed at the Johns Hopkins Bloomberg School of Public Health, manages TCI's implementation through five regional hub partners: East Africa: Jhpiego; Francophone West Africa: IntraHealth International; India: Population Services International (PSI); Nigeria: Johns Hopkins Center for Communication Programs (CCP); and the Philippines: the Zuellig Family Foundation (ZFF). Currently, TCI supports 104 cities in implementing high-impact interventions for family planning and AYSRH. Of these, 39 have a distinct focus on AYSRH programming and cover a total population of nearly 45 million (Figure 1 and Table 1).

**Table 1:** TCI geographic and population coverage.

**Country**	**Cities/States**	**Total population**	**Youth 15–24**
Benin	Cotonou, UCOZ	1,804,696	204,369
India	Agra, Aligarh, Allahabad, Bareilly, Faizabad, Firozabad, Ghaziabad, Gorakhpur, Kanpur, Lucknow, Mathura, Meerut, Saharanpur, Shahjahanpur, and Varanasi	19,232,784	1,639,542 (Focus on First-Time Parents)
Kenya	Malindi, Migori, Mombasa, Kamukunji, Kasarani, Ruraka, Nyamira, and Vihiga	6,431,492	806,228
Nigeria	Edo, Niger, Ogun, and Plateau	10,007,753	948,317
Senegal	Ziguinchor	662,473	64,677
Tanzania	Arusha DC, Arusha City, Ilala, Kinondoni, Tanga City, and Geita	4,455,337	496,730
Uganda	Buikwe, Iganga, and Mukono	1,947,667	204,969
TOTAL	39 cities/states	44,542,202	4,364,832

*Estimates made using census and UN Population Division data*.

TCI's goal is greater self-reliance of city governments to scale up family planning and AYSRH high-impact interventions, leading to sustained improvements in urban health systems and increased use of modern contraception, especially among the urban poor. Its main focus has been on strengthening city governments' capacity to implement high-impact interventions for family planning in a sustainable way. TCI lets city governments lead their own family planning and AYSRH programming for scale, impact, and sustainability. Cities self-select to participate in TCI, committing their own financial and human resources, and political will. As a participant, cities can access TCI's Challenge Fund, which incentivizes participation with seed funding from donors, including foundations, the private sector, and private philanthropists. Cities identify their own family planning and AYSRH program needs and TCI provides technical support with its high-impact interventions housed in an online learning platform called TCI University (TCI-U). Cities also receive guidance from experienced family planning/AYSRH experts—or coaches—to adapt, apply, scale, and sustain these interventions.

### A Heightened Focus on AYSRH

In June 2018, TCI was given additional funding by the Gates Foundation to heighten its focus on improving contraceptive access for adolescents and youth 15–24 years of age^1^—within the larger cohort of women of reproductive age (WRA) 15–49 years of age already supported by its family planning program. This was in response to demands from stakeholders at the local level concerned about increasing unintended pregnancies among adolescents. The additional funding allowed TCI to dedicate technical and program support to married (including first-time parents) and unmarried youth in TCI-supported cities.

The following primary outcomes are part of TCI's results framework:

- Increased number of local governments effectively implementing family planning and AYSRH interventions.
- Increased uptake of modern contraceptive methods among women ages 15–49, with an increased emphasis on the urban poor and youth 15–24 years.
- Improved local government leadership and ownership in implementing effective and sustainable family planning and AYSRH programs.

While government leaders may be committed to improving contraceptive access for young people, many lack clear guidance on how to do so effectively (23). TCI's AYSRH technical and program experts share such guidance with local governments, build upon interventions that have proven effective, and support any new promising evidence in AYSRH programs to demonstrate results quickly. Twelve proven AYSRH interventions are curated in a global AYSRH-specific toolkit that launched on TCI-U in 2018. From these original 12 interventions, TCI's five hubs have developed 31 related AYSRH interventions that were adapted from the global toolkit for their local context. For example, the global toolkit's Adolescent and Youth-Friendly Services' intervention has locally adapted versions in the East Africa, Francophone West Africa, Nigeria, and Philippines' toolkits.

### How TCI Engages Urban Slums

Cities submit formal expressions of interest (EOI) detailing their political commitment to family planning or AYSRH, resource contribution, health systems readiness, as well as the size of potential population impact. TCI has received 82 EOIs to date for AYSRH programming. Once TCI approves an EOI, the city and TCI co-develop a program design that includes a landscaping exercise and gap analysis to identify challenges that can be addressed by selecting appropriate interventions in TCI-U. TCI-U's dynamic learning platform ensures the high-impact interventions are constantly updated with learnings from real-world experience.

When TCI added AYSRH in East Africa, Francophone West Africa, India and Nigeria in 2018, it applied the same demand-driven model as outlined above and cities (or states in the case of Nigeria) were selected to receive AYSRH-focused coaching along with the TCI Challenge Fund.

In Nigeria, for example, after seven states submitted EOIs, Niger, Ogun, Plateau, and Edo states were selected. They were then supported to complete program designs while committing resources to implement AYSRH interventions. During the program design phase, 29 urban and peri-urban Local Government Areas (LGAs) were selected based on population size. A total of 130 high-volume health facilities were selected for scale up in the 29 LGAs.

In India, a more elaborate mapping process occurs for urban slums. It is difficult to assess urban boundaries so demarcating the slum area is a significant first step for any successful urban intervention. TCI developed a mapping and listing tool that identifies residents in need of services by determining the number of people living in slums and what is needed to meet their health needs. For example, this exercise can determine how many Auxiliary Nurse Midwives (ANMs), accredited social health activists (ASHAs), and urban primary health centers (UPHCs) are necessary to serve this population. In addition, the Uttar Pradesh government provides all ASHAs with registry diaries to maintain health records on the women, men, and children in their catchment areas. The diary records immunizations, antenatal and prenatal care, institutional delivery, family planning, and other health service areas. Systematically maintaining this data is crucial for ensuring those with various health needs are counseled by ASHAS and provided information and referrals to services. This is especially important because ASHAs can identify first-time parents and counsel them on available family planning services based on their need and choice.

### TCI's Concentric Circle Strategy Helps AYSRH Programming Go to Scale

**Figure 2:**
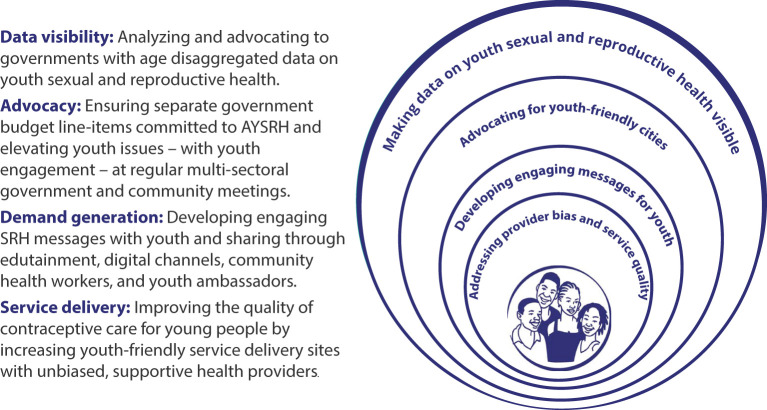
TCI's concentric circle strategy for AYSRH programming.

TCI's concentric circle AYSRH strategy (Figure 2) is the framework guiding its support to city governments to improve access to contraception for youth in urban slums. It emphasizes as a top priority the importance of making AYSRH data visible to decision-makers, community members, and youth themselves. It highlights the importance of youth engagement with government and community leaders to advocate for youth sexual and reproductive health (SRH) issues including funding. Segmented demand generation messages for youth and their influencers (i.e., partners, parents, teachers, and other gatekeepers) are also essential through both interpersonal communication, WhatsApp chatrooms, and media channels. And central to the strategy is addressing provider bias and service quality so youth are linked to quality AYSRH services delivered by sensitized facility staff oriented to set aside their biases against contraceptive provision—in particular for unmarried youth. The implementation process for this strategy is described below.

### Making Data on Youth Sexual and Reproductive Health Visible

Most governments only report contraceptive uptake for women ages 15–49 without age disaggregation. Uganda and Senegal are currently the only TCI-supported countries whose health management information systems (HMIS) disaggregates data on contraceptive use by age and method in all facilities. While TCI has observed early, promising results from its AYSRH implementation in Uganda, more study is needed to determine the extent of TCI's impact there. TCI supports city governments in collating data on youth contraceptive use directly from health facility registers. It then coaches governments on using this data to monitor impact and, importantly, advocate to local, state and national government officials to prioritize data collection, funding, and programming for youth.

In Nigeria, TCI helped advocate for a national HMIS tool with age-disaggregation to include adolescent age groups with 5-year age intervals. This would allow tracking for all conditions for which youth attend public health facilities, including contraceptive use. Likewise in India, TCI improved the use of an existing HMIS form to better track adolescent use of public health facilities.

### Advocating for Youth-Friendly Cities

TCI advocates for increased government investment and leveraging of existing funding for AYSRH. TCI-supported city governments release resources for improving youth contraceptive access and underlying AYSRH conditions and needs. TCI also supports governments to integrate the engagement of youth and relevant civil society organizations in regular meetings—such as ministry of health meetings and multisectoral working groups. In Francophone West Africa, TCI-supported Jeunes Leaders Transformationnels (JLT) are youth ages 15–24 advocating to government and community leaders about AYSRH. In East Africa, TCI helps governments identify and coach youth champions ages 15–24 to become community advocates including implementing intergenerational discussions on AYSRH, conducting demand generation activities, and engaging in governance- level discussions. In Nigeria, TCI works with state and local governments to identify and coach a diverse group of young people ages 15–35 as Life Planning for Adolescents and Youth (LPAY) ambassadors. These ambassadors are embedded in regular governance and community structures to elevate youth SRH needs, including technical working groups on adolescent health hosted by the state. In India, TCI supported the launch of AYSRH programs in cities through coordination workshops organized and managed by youth and attended by the National Health Mission as well as youth functionaries from state and municipal government.

### Developing Engaging Messages for Youth and Their Influencers

Urban youth have intersecting identities that impact their SRH behaviors, and these behaviors are related to their age, marital status, education attainment, economic status, religion, and parenthood. TCI coaches governments to identify youth sub-groups most in need and tailor approaches for effective reach with messages and resources for greatest impact. In India, where sexual debut is principally within marriage, TCI focused initially on first-time parents to improve contraceptive use among married young women with recent births. In Benin, Kenya, Senegal, Tanzania, and Uganda, TCI focused on unmarried youth because of high unintended pregnancy rates. TCI partnerships aim to decrease too-early, unplanned pregnancies that imperil health, while derailing education and life plans. In Nigeria, TCI and state governments primarily focus on reaching unmarried youth as well, but also recognizes cultural sensitivities in more conservative states where married youth are also a focus.

TCI helps cities strengthen the capacity of community health workers to reach young people with contraceptive information, referrals to health facilities, and other events for contraceptive methods. These community health workers are also coached on providing contraceptive counseling to clients based on age, marital status, and parity. TCI's demand generation work also engages youth with influencers who can control their access to contraceptives, such as religious leaders, parents, teachers, and male partners. In Nigeria and East Africa, TCI coaches local government stakeholders, youth leaders, and civil society organizations to host community dialogues for such influencers to discuss AYSRH issues leading up to an AYSRH service day in a nearby health facility.

### Addressing Provider Bias and Service Quality

A core component of TCI's strategy of scaling high-impact AYSRH interventions is supporting governments to address provider bias toward youth contraceptive use in a sustainable way. TCI helps cities conduct technical and values-clarification exercises with health providers, equipping them with the knowledge and tools to provide non-judgmental, attitudinally respectful, and supportive care to young people (24, 25). In addition, TCI supports cities in conducting AYSRH whole-site orientations in facilities to sensitize all staff on young people's needs, including administrative and security staff (26). TCI also supports governments to improve the quality of youth-friendly health services through application of national checklists during routine supervisory visits (27).

In East Africa, TCI cities hold youth-focused integrated outreaches (community events) and in-reaches (in facility events) to increase access to contraceptives. In Francophone West Africa, TCI supports city governments in holding free family planning days for youth, where they can access free contraceptives. In India, ASHAs in TCI cities refer all first-time parents in their catchment areas to monthly Fixed-Day Services (FDS) for contraceptive methods at UPHCs. In addition to holding AYSRH whole-site orientations, TCI conducts social mobilization events to reach youth in Nigeria with referrals to youth-friendly health facilities.

### Working With Private-Sector Pharmacies to Reach Youth

Condoms are a preferred choice of contraceptives for youth and pharmacies are preferred sources of condoms and other short-term contraceptive methods (including emergency contraception). TCI is helping make pharmacies more youth friendly in East Africa, Francophone West Africa, Nigeria, (28) and just starting in India. In Kenya, Tanzania, and Uganda, TCI is engaging pharmaceutical and drug shop associations to not only increase access to contraceptive services by young people but also improve the knowledge, attitudes, and skills of pharmacists and drug shop operators on how to deliver adolescent and youth-friendly services (29). The pharmacists and drug shop operators have been oriented on national guidelines for family planning and youth-friendly services, how to make referrals to nearby public facilities, and the importance of documentation and reporting on services provided as well as referrals made.

### A Typical Process for High-Impact AYSRH Programming

Once a city's program design is approved, TCI supports activities simultaneously at the governance, health facility, and community levels with active youth engagement. The gap analysis conducted during the program design likely uncovered scarce age-disaggregated data for youth. Addressing this requires strategic advocacy for committed budget lines for AYSRH, including completion of staffing rosters during monthly or quarterly governance-level meetings. Youth engagement in these meetings are gradually ensured through community-level identification and coaching of relevant youth. The minutes from these meetings are tracked for commitments and fulfillment, which are monitored in regular TCI reporting.

At the facility level, specific staff are identified for offsite training using the government's AYSRH curriculum—but modified with high-impact interventions and tools from TCI-U. Once offsite training is completed, whole-site orientations are conducted with the entire facility staff from the Medical Officer-in-Charge to the cleaning staff. This ensures respectful, non-judgmental, and supportive services that adhere to the government's quality improvement/assurance (QI/QA) checklists are implemented. TCI also helps review checklists in accordance with national adolescent health standards and WHO Guidelines on adolescent and youth-friendly health services. Checklist results are monitored for improvement on an annual basis with actions taken to address identified problems. TCI continues to track these on a regular basis. In addition, TCI supports the government's community outreach functionaries through orientation on TCI-U materials for generating community-level support for AYSRH. Advocacy and dialogue are supported with gatekeepers, with youth engagement, and interactions with existing community organizations that support health (e.g., women's clubs, male involvement, and community theater).

At the community level, TCI catalyzes demand generation from youth with support of peers, parents, and other gatekeepers in their communities. The key functionaries involved are community health workers and active youth groups supported by the Ministry of Youth using tools from TCI-U *via* face-to-face, print, and WhatsApp. TCI also monitors these steps regularly.

### Assessing Performance and Adapting Accordingly

In addition to the performance monitoring outlined above, TCI's graduation strategy is part of its core principle of sustaining local financing and ownership to ensure continued leadership and city-led implementation of family planning and AYSRH programs. TCI's coaching and Challenge Fund support gradually diminishes as cities demonstrate increased capacity. City engagement with TCI ultimately culminates in a move toward greater self-reliance as a city takes strategic steps to graduate from TCI.

TCI uses data to inform local government problem-solving and decision-making, including HMIS, project records, population-based surveys, and qualitative methods such as the Most Significant Change (MSC) technique. TCI's Reflection and Action to Improve Self-reliance and Effectiveness (RAISE) performance assessment tool has specific criteria related to available data and milestones to help local governments evaluate the quality and effectiveness of their activities.

**Figure 3:**
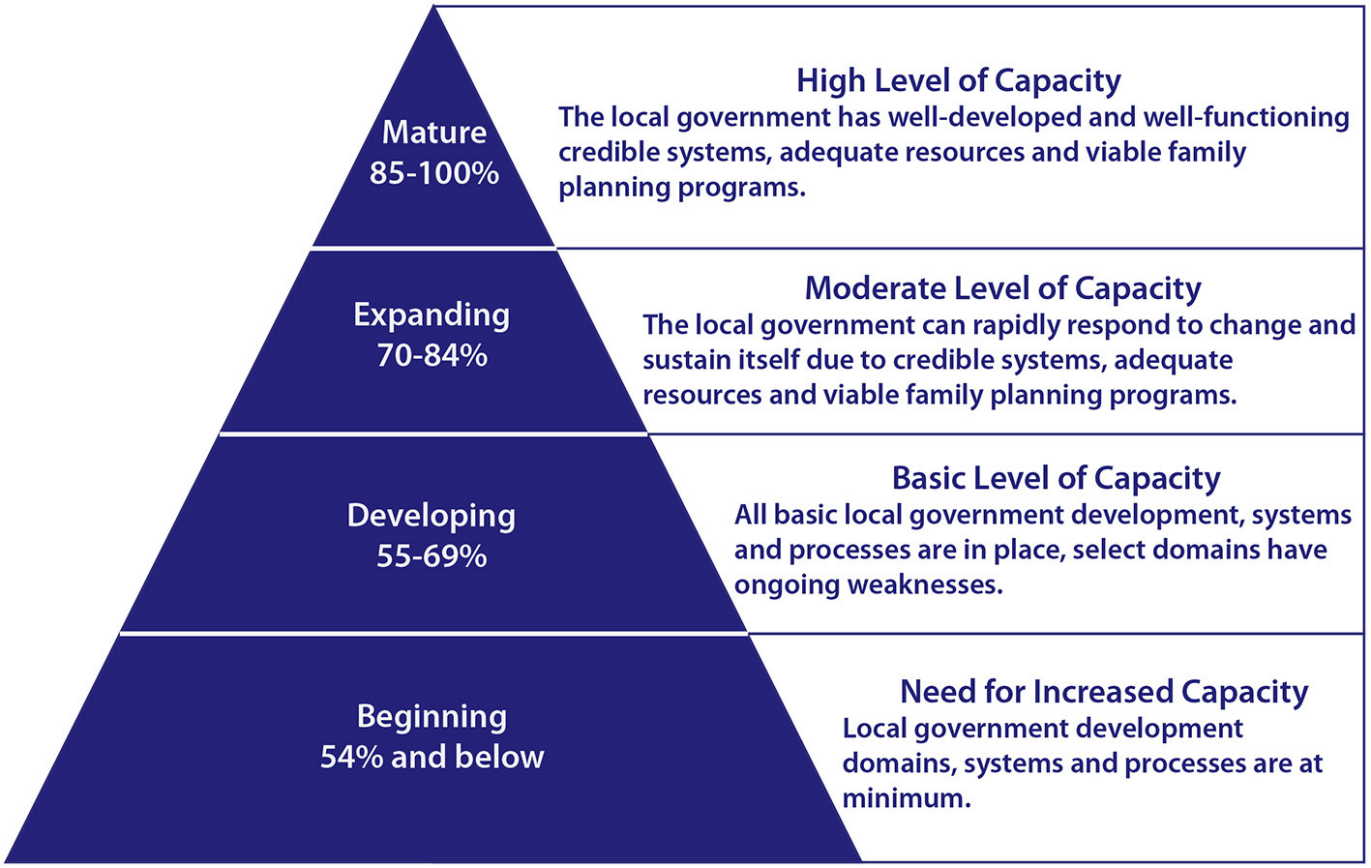
Four levels of performance are possible when conducting a RAISE assessment.

Each quarter, a city's key health personnel evaluate the quality and effectiveness of their activities and implementation strength to make necessary course corrections. At RAISE assessment workshops, participants review relevant external data to validate their scoring. As participants work to reach consensus on scores, they provide evidence in the form of policy documents, program reports, budgets and expenditure reports. A score of 85% or better indicates a high-level of capacity (70–84% is a moderate level of capacity, 55–69% is a basic level and anything below 54% shows a need for increased capacity) (Figure 3). Both local health management teams and TCI use RAISE results to track the level of city readiness toward graduation along a 3-year continuum to achieve performance milestones.

With the RAISE tool as the primary data source, TCI assesses performance, identifies governments ready for graduation, and starts to engage government leaders to develop a data-informed graduation plan. So far, 25 cities implementing family planning interventions have graduated from TCI, but no AYSRH cities have graduated yet. The graduated cities' RAISE scores have held steady so far or risen in a few cases.

## Results

Table 2 details the results of two rounds of RAISE assessments related to AYSRH programming from selected cities. In all cases, scores increased from Round 1 to 2 due to targeted coaching from TCI on low-ranking areas in Round 1.

**Table 2:** RAISE assessments for six TCI cities and actions taken leading to improvements.

**Location**	**RAISE scores** **by round**	**Program activities**	
		**Key areas contributing to increases**	**Coaching activities contributing to increases**
**KENYA**Mombasa	Round 1: 80% Round 2: 92% (+12%)	• Financial commitment for AYSRH • Supportive supervision • Coaching • Community involvement • Adolescent and youth-friendly services	• Continuous political engagement • Continuous advocacy for more resources • Procure adequate tools • Referral and linkages • Follow up of coaches • Avail and print guidelines for volunteers
**TANZANIA**Ubungo	Round 1: 81% Round 2: 89% (+8%)	• Advocacy • TCI-U access and utilization • Coaching • Supportive supervision • Public-private partnership	• Use of FP champions for advocacy • Enroll and orient more TCI-U users • Conduct supportive Supervision • Partnership with private sector, i.e., pharmacies and other NGOs providing FP/AYSRH services
**UGANDA**Mukono	Round 1: 48% Round 2: 88% (+40%)	• Financial documentation and management • Family planning /AYSRH strategies/approaches • Coaching • Supportive supervision	• Non-technical coaching on financial management and documentation • Disseminate family planning/AYSRH best practices at all levels • Sisi-kwa-sisi coaching • Supportive supervision
**FWA**Ziguinchor, Senegal	Round 1: 43% Round 2: 74.5% (+31.5%)	• Strengthened collaboration between municipality and health system • AYSRH effectively layered onto TCI family planning program • Strong landscaping to identify gaps and needs • Strong political and health system commitment to AYSRH	• Supportive supervision for AYSRH • Implementation and monitoring of AYSRH best practices, including reduction of provider bias, whole-site orientation, comprehensive sexual education, home visits by community health workers, youth associations, social media, and the transformational youth leaders • Adolescent and youth-friendly checklist operationalized
**INDIA**Saharanpur	Round 1: 66% Round 2: 73% (+7%)	• Improved leadership for AY interventions • Youth participation in key meetings • Review of AY program at city coordination committee meetings • City leading AYFHS assessments • Improved referral system • Frontline health workers map and list adolescents and refer to UPHCs • Advocacy for inclusion of AY indicators and data from HMIS in review meetings	• Management coaching facility staff for timely upload of AY data on HMIS portal • Coaching LG to review data from facility and community at monthly review meetings • Follow up of master coaches • Re-stock and supplies (condoms, OCP, and EC) • Supportive supervision of AY counselors at District Hospitals and District Women's Hospitals • Partnership with private sector, including pharmacies
**NIGERIA**Edo State	Round 1: 53% Round 2: 69% (+16%)	• State adoption and scale up of best practices • Increased social mobilization and referrals for AY services • Improved provider behavior and increased availability of AYFHS	• Sensitizing policymakers on need for dedicated AYSRH programming and funding • Advocacy to create a budget line for AYSRH • Coach state team on scale up of best practices and train providers in AYFHS

## Discussion

In just 3 years, TCI's platform has institutionalized a systems-strengthening strategy with city governments adding AYSRH interventions to family planning programming to improve youth access to contraception in urban slums. It did this across 24 socio-culturally diverse cities in sub-Saharan Africa and 15 cities in Uttar Pradesh by supporting activation of both public and private health systems, their community networks, and youth organizations. TCI's demand-driven approach ensured city government commitment, catalyzed by their contributions, with partnerships between municipal and health systems and youth organizations to facilitate community-level support for AYSRH in the urban space. Rapid scale-up of TCI interventions was facilitated by ease of access to and coaching on evidence-based interventions and “how-to” tools in TCI-U's AYSRH toolkit. Assessments using the RAISE tool identified what areas needed more support or coaching, leading to improved performance validated by repeat assessments. Of note, the RAISE assessments flagged the areas for improvement to include supportive supervision, public-private partnerships, adolescent and youth-friendly health services, and ensuring method mix.

## Study Limitations

The lack of age-disaggregated facility data on clients and the absence of surveys hindered impact assessment on contraceptive uptake by method and measures of universal contraceptive services coverage.

## Conclusion and Recommendations

TCI's AYSRH systems-strengthening approach grounded in high-impact interventions rapidly scaled to 39 cities and multiple urban slums. TCI supported segmented demand generation with youth engagement, improving access to quality and affordable contraceptives and AYFHS. Its menu of interventions—including public-private partnerships with pharmacies and quality assurance in facilities using quick checklists—use an innovative coaching model and engage with youth as partners. This approach has improved youth access to reliable long- and short-acting modern contraceptive methods. Youth engagement at governance levels and with their communities added a layer of accountability as clients, while the RAISE tool brought in partner perspectives on achievement. In this decade of universal health coverage, it is imperative to complete assessments of sufficient coverage of quality contraceptive services for adolescents and youth. It will be necessary to triangulate a sample of age-disaggregated data from public and private facilities and pharmacies and/or conduct a population-based survey with comparison sites for both methods. The survey will have the added advantage of describing the pathways from information provision to ideation to becoming a contraceptive user with method choice.

## Data Availability Statement

The original contributions generated for the study are included in the article/Supplementary Material, further inquiries can be directed to the corresponding author/s.

## Author Contributions

KB, KM, and KW contributed to conception and design of the study. PN, MLS, AB, DS, and TK contributed to East Africa data and text. HT and JA contributed to Francophone West Africa data and text. MS and DV contributed to India data and text. VI and DA contributed to Nigeria data and text. MM, KG, VM, and AF reviewed and edited data and text from East Africa, Francophone West Africa, India, and Nigeria. KB and KM wrote the first draft of the manuscript. All authors read, revised, and approved the submitted version.

## Funding

This study was funded by the Bill & Melinda Gates Foundation (OPP1145051). Bayer AG (JHU Grant No. 123709), Comic Relief (JHU Grant No. 4581036).

## Conflict of Interest

The authors declare that the research was conducted in the absence of any commercial or financial relationships that could be construed as a potential conflict of interest.

## Publisher's Note

All claims expressed in this article are solely those of the authors and do not necessarily represent those of their affiliated organizations, or those of the publisher, the editors and the reviewers. Any product that may be evaluated in this article, or claim that may be made by its manufacturer, is not guaranteed or endorsed by the publisher.

## Footnotes

^1^TCI uses the United Nations' definition for youth: 15 to 24 years, endorsed by the UN General Assembly in its resolution 36/28 of 1981.
