# Supplementary material for: Scaling Access to Contraception for Youth in Urban Slums: The Challenge Initiative's Systems-Based Multi-Pronged Strategy for Youth-Friendly Cities
Source: Front Glob Womens Health. 2021 Oct 1;2:673168. doi: 10.3389/fgwh.2021.673168 (PMC8597915; doi:10.3389/fgwh.2021.673168)
Supplement: Supplementary file 2 [file Table_2.DOCX]

| **Location** | **RAISE Scores by Round** | **Program Activities** | |
| --- | --- | --- | --- |
|  |  | **Key areas contributing to increases** | **Coaching activities contributing to increases** |
| KENYA  Mombasa | Round 1: 80% | - Financial commitment for AYSRH - Supportive supervision - Coaching - Community involvement - Adolescent and youth-friendly services | - Continuous political engagement - Continuous advocacy for more resources - Procure adequate tools - Referral and linkages - Follow up of coaches - Avail and print guidelines for volunteers |
|  | Round 2: 92% (+12%) |  |  |
| **TANZANIA**  Ubungo | Round 1: 81% | - Advocacy - TCI-U access and utilization - Coaching - Supportive supervision - Public-private partnership | - Use of FP champions for advocacy - Enroll and orient more TCI-U users - Conduct supportive Supervision - Partnership with private sector, i.e., pharmacies and other NGOs providing FP/AYSRH services |
|  | Round 2: 89% (+8%) |  |  |
| **UGANDA**  Mukono | Round 1: 48% | - Financial documentation and management - Family planning /AYSRH strategies/approaches - Coaching - Supportive supervision | - Non-technical coaching on financial management and documentation - Disseminate family planning/AYSRH best practices at all levels - Sisi-kwa-sisi coaching - Supportive supervision |
|  | Round 2: 88% (+40%) |  |  |
| **FWA**  Ziguinchor, Senegal | Round 1: 43% | - Strengthened collaboration between municipality and health system - AYSRH effectively layered onto TCI family planning program - Strong landscaping to identify gaps and needs - Strong political and health system commitment to AYSRH | - Supportive supervision for AYSRH - Implementation and monitoring of AYSRH best practices, including reduction of provider bias, whole-site orientation, comprehensive sexual education, home visits by community health workers, youth associations, social media and the transformational youth leaders - Adolescent and youth-friendly checklist operationalized |
|  | Round 2: 74.5%  (+31.5%) |  |  |
| **INDIA**  Saharanpur | Round 1: 66% | - Improved leadership for AY interventions - Youth participation in key meetings - Review of AY program at city coordination committee meetings - City leading AYFHS assessments - Improved referral system - Frontline health workers map and list adolescents and refer to UPHCs - Advocacy for inclusion of AY indicators and data from HMIS in review meetings | - Management coaching facility staff for timely upload of AY data on HMIS portal - Coaching LG to review data from facility and community at monthly review meetings - Follow up of master coaches - Re-stock and supplies (condoms, OCP and EC) - Supportive supervision of AY counsellors at District Hospitals and District Women’s Hospitals - Partnership with private sector, including pharmacies |
|  | Round 2: 73% (+7%) |  |  |
| **NIGERIA**  **Edo State** | Round 1: 53% | - State adoption and scale up of best practices - Increased social mobilization and referrals for AY services. - Improved provider behavior and increased availability of AYFHS | - Sensitizing policymakers on need for dedicated AYSRH programming and funding - Advocacy to create a budget line for AYSRH - Coach state team on scale up of best practices and train providers in AYFHS. |
|  | Round 2: 69% (+16%) |  |  |
